# The Laughter Cure

**Published:** 1899-08

**Authors:** 


### THE LAUGHTER CURE.

We have all heard of the patient who, while nearly suffocating under a bad attack of quinsy, laughed so hard at some ludicrous incident, that the abscess broke and he recovered forthwith. Dr. Russell H. Conwell, the noted Philadelphia divine, in his popular lecture, "The Jolly Earthquake," illustrating the value of laughter as a therapeutic agency, tells about a similar but far more remarkable occurrence, as follows:

"A man was suffering from that most dreaded of all diseases, hydrophobia. Attendants held him to the bed, that he might do no harm to himself or to other members of the family. The death dew was already on his forehead and the family had been called in to witness the last sad agony. His sister, in her excitement, brought with her to the room her little three-year-old boy, not noticing what he carried with him. At the bedside the little fellow, wishing to contribute as best he could to make his uncle's passage a peaceful and happy one, held up a gayly-painted jumping-jack, working it most energetically. The poor sufferer, in a lucid interval, catching sight of this rather novel form of entertainment at the deathbed, began to laugh, laughed long and loud, and at last exhausted, sank into a state of deep unconsciousness from which he soon awakened asking for water. The danger was over. The disease had succumbed to a laugh. Dr. Clark, of Bellevue, who first related the story, adds: 'It would be better for many patients if they had jumping-jacks for their doctors.' "—*Medical Times.*

